# Exon Silencing by UAGG Motifs in Response to Neuronal Excitation

**DOI:** 10.1371/journal.pbio.0050036

**Published:** 2007-02-06

**Authors:** Ping An, Paula J Grabowski

**Affiliations:** Department of Biological Sciences, University of Pittsburgh, Pittsburgh, Pennsylvania, United States of America; University of Wisconsin, United States of America

## Abstract

Alternative pre-mRNA splicing plays fundamental roles in neurons by generating functional diversity in proteins associated with the communication and connectivity of the synapse. The CI cassette of the NMDA R1 receptor is one of a variety of exons that show an increase in exon skipping in response to cell excitation, but the molecular nature of this splicing responsiveness is not yet understood. Here we investigate the molecular basis for the induced changes in splicing of the CI cassette exon in primary rat cortical cultures in response to KCl-induced depolarization using an expression assay with a tight neuron-specific readout. In this system, exon silencing in response to neuronal excitation was mediated by multiple UAGG-type silencing motifs, and transfer of the motifs to a constitutive exon conferred a similar responsiveness by gain of function. Biochemical analysis of protein binding to UAGG motifs in extracts prepared from treated and mock-treated cortical cultures showed an increase in nuclear hnRNP A1-RNA binding activity in parallel with excitation. Evidence for the role of the NMDA receptor and calcium signaling in the induced splicing response was shown by the use of specific antagonists, as well as cell-permeable inhibitors of signaling pathways. Finally, a wider role for exon-skipping responsiveness is shown to involve additional exons with UAGG-related silencing motifs, and transcripts involved in synaptic functions. These results suggest that, at the post-transcriptional level, excitable exons such as the CI cassette may be involved in strategies by which neurons mount adaptive responses to hyperstimulation.

## Abstract 2

Exon skipping of NMDA R1 receptor exon 21 (CI cassette) is mediated by UAGG-type silencing motifs, presenting a mechanism by which external stimuli communicate to the nuclear splicing machinery in neurons.

## Author Summary

The modular features of a protein's architecture are regulated after transcription by the process of alternative pre-mRNA splicing. Conditions that excite or stress neurons can induce changes in some splicing patterns, suggesting that cellular pathways can take advantage of the flexibility of splicing to tune their protein activities for adaptation or survival. Although the phenomenon of the inducible splicing switch (or inducible exon) is well documented, the molecular underpinnings of these curious changes have remained mysterious.

We describe methods to study how the glutamate NMDA receptor, which is a fundamental component of interneuronal signaling and plasticity, undergoes an inducible switch in its splicing pattern in primary neurons. This splicing switch promotes the skipping of an exon that encodes the CI cassette protein module, which is thought to communicate signals from the membrane to the cell nucleus during neuronal activity. We show that this induced splicing event is regulated in neurons by a three-part (UAGG-type) sequence code for exon silencing, and demonstrate a wider role for exon-skipping responsiveness in transcripts with known synaptic functions that also harbor a similar sequence code.

## Introduction

Alternative pre-mRNA splicing expands protein functional diversity by directing precise nucleotide sequence changes within mRNA coding regions. Splicing regulation often involves adjusting the relative levels of exon inclusion and skipping patterns as a function of cell type or stage of development. In the nervous system, such changes affect protein domains of ion channels, neurotransmitter receptors, transporters, cell adhesion molecules, and other components involved in brain physiology and development [1,2].

There is growing evidence that various biological stimuli, such as cell excitation, stress, and cell cycle activation, can induce rapid changes in alternative splicing patterns [3,4]. These phenomena suggest that splicing decisions may be altered by communication between signal transduction pathways and splicing machineries, but such molecular links and mechanisms are largely unknown. The focus of the present study is to gain insight into these mechanisms using primary neurons as the model system.

Splicing decisions take place in the context of the spliceosome, which is the dynamic ribonucleoprotein machinery required for catalysis of the RNA rearrangements associated with intron removal and exon joining [5–7]. Spliceosomes assemble on pre-mRNA templates by the systematic binding of the small nuclear ribonucleoprotein particles, U1, U2, and U4/U5/U6, which leads to splice site recognition and exon definition. Thus, splicing decisions can be profoundly influenced by the strength of the individual 5′ and 3′ splice sites and by auxiliary RNA sequences that tune splice site strength via enhancement or silencing mechanisms. RNA binding proteins from the serine/arginine-rich (SR) and heterogeneous nuclear ribonucleoprotein (hnRNP) families play key roles in recognizing auxiliary RNA sequences from sites within the exon (exonic splicing enhancers or silencers; ESEs or ESSs, respectively) or intron (intronic enhancers or silencers; ISEs or ISSs, respectively). Despite numerous RNA motifs that have been functionally characterized as splicing enhancers or silencers, the mechanisms by which these motifs function in combination to adjust splicing patterns are not yet well understood [8,9]. The broad significance of this problem is highlighted by the observation that nearly 75% of human pre-mRNAs with multiple exons undergo alternative splicing [10]. In addition, numerous point mutations in splice sites or splicing control elements have been linked to genetic disease [11].

Insights into the molecular basis for induced effects on alternative splicing have come from a variety of biological systems. Cell excitation by KCl-induced membrane depolarization in the pituitary cell line, GH3, has been shown to induce skipping of several cassette exons, including the STREX exon of the BK slo channel, as well as the N1 and C1 exons of the glutamate NMDA receptor NR1 subunit (*GRIN1* gene) [12]. Note that the CI cassette exon is called exon 21 in other studies. Calcium/calmodulin kinase (CaMK) IV was implicated in these effects based on the loss of excitation-induced exon skipping by a specific inhibitor of this enzyme, whereas the reverse effect was observed by expression of the catalytic subunit of the enzyme. In this study, a 54-nucleotide region of the 3′ splice site of the STREX exon was identified and found to be sufficient to mediate similar effects when attached to a heterologous exon. In another study, application of the drug pilocarpine induced skipping of the EN exon of *clathrin light chain B,* the N1 exon of c*-src,* and the CI exon of *GRIN1* transcripts in the rat hippocampus and/or cortex of living animals [13]. In the T cell–derived cell line JSL1, phorbol ester treatment has been shown to induce cell excitation together with increased skipping of exon 4 of CD45 pre-mRNA. These effects are dependent upon a 60-nucleotide splicing silencer within exon 4, and hnRNP L has been implicated as a factor involved in mediating these effects [14,15]. In another study, phorbol ester has been shown to increase CD44 v5 exon inclusion in a manner that was dependent upon Sam68 and the MAPK/ERK pathway [16]. In HeLa cells, the cellular response to heat shock was found to generate potent and widespread splicing silencing, and SRp38 was identified as the protein factor responsible for these effects [17]. A general silencing effect on pre-mRNA splicing by SRp38 was also observed in cells undergoing mitosis [18].

Recent studies have implicated hnRNP A1 as a factor that mediates induced changes in alternative splicing in response to osmotic stress. NIH 3T3 cells that have undergone osmotic stress have a relative increase in cytoplasmic A1 compared to nuclear A1, and this effect is associated with the phosphorylation of carboxy terminal residues that extend into the M9 region [19,20]. HnRNP A1 has been shown to regulate a variety of alternative splicing events through the recognition of RNA sequences related to the consensus binding motif, UAGGGA/U [11,21,22]. Two RNA binding domains and a glycine-rich region are involved in sequence-specific recognition, and a sequence near the carboxyl terminus, M9, is required for nuclear import. HnRNP A1 has been shown to function as a substrate for protein kinase C (PKC), protein kinase A (PKA), and casein kinase [23]. HnRNP A1′s ability to silence splicing of CD44 v5 exon was reduced by the overexpression of a constitutively active form of the mitogen-activated protein/ERK kinase kinase 1, and by the small GTP-binding protein Cdc42 in NIH 3T3 and erythroleukemia cell lines [24]. Together, these results suggest that the functional properties of hnRNP A1 could be altered in complex ways through signaling pathways in response to various biological stimuli.

The CI cassette exon of the *GRIN1* gene was chosen for this study based on its known hnRNP A1–mediated exon silencing mechanism, which involves two exonic UAGGs and an intronic GGGG motif [25]. Here we develop a system to study the response of the CI cassette to cell excitation in neurons of primary cultures, and we utilize this system to investigate the role of the UAGG-related exon silencing code in the induced exon-skipping response. We extend this with complementary biochemical approaches to test for binding- and expression-level effects on hnRNP A1 protein in nuclear extracts prepared from resting and excited cultures. We also take advantage of the primary neuronal cultures to explore the role of endogenous ion channels in the induced exon-skipping phenomenon using antagonists of the NMDA receptor and other physiologically relevant calcium channels. An expanded analysis of exon-skipping responsiveness of endogenous transcripts involved in synaptic and non-synaptic functions is also presented.

## Results

### Assay for Activity-Induced Alternative Splicing in Primary Neurons and Glia Using Reporters Driven by Cell-Specific Promoters

Primary neurons, due to their responsiveness and plasticity, should represent a useful model system to study activity-induced changes in alternative splicing, but to date, such systems have not been well developed. In this study, we have developed low-density cortical cultures from the embryonic rat forebrain as an experimental system to address the molecular mechanisms of induced changes in alternative splicing using cell-specific and biochemical approaches. Upon dissociation and plating in culture, the postmitotic neurons extend processes, establish synaptic connections, and become electrically active, whereas glial cells in these cultures play supportive roles.

Initially, changes in endogenous splicing patterns were monitored in the cortical cultures both as a function of KCl-induced depolarization, and cell differentiation using the CI cassette exon as a readout. The cortical cultures at 6, 8, 12, and 14 d in vitro (DIV) after plating were treated for 24 h with media containing 25 or 50 mM KCl, since these conditions are known to induce membrane depolarization in these cultures. Changes in the percent exon inclusion values were calculated as the difference between the value in the 50 mM KCl sample and the mock-treated control (ΔEI values).

**Figure 1:**
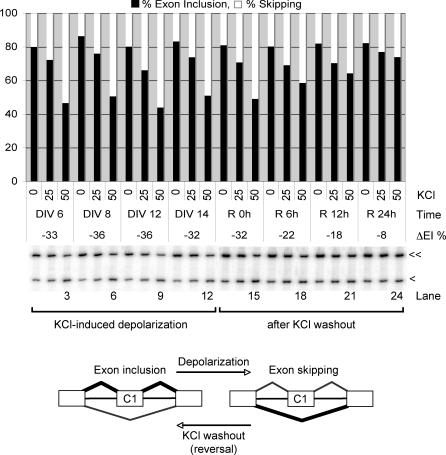
Reversibility and Effects of Neuronal Differentiation in the Response of the CI Cassette Exon to KCl-Induced Depolarization in Rat Cortical Cultures Alternative splicing of the endogenous CI cassette exon (NMDA R1 transcript) was monitored in rat cortical cultures in response to KCl treatment beginning at day 6 (lanes 1–3), 8 (lanes 4–6), 12 (lanes 7–9), and 14 (lanes 10–12) after plating. Cell cultures were treated with 0, 25, or 50 mM KCl in the culture medium for 24 h. Reversibility of induced effects was monitored in day 12 cultures, which were treated with 0, 25, and 50 mM KCl for 24 h followed by washout into fresh medium without KCl for an incubation time of 0 (lanes 13–15), 6 (lanes 16–18), 12 (lanes 19–21), and 24 (lanes 22–24) h after washout. Relative quantities of exon-included (<<) and -skipped (<) mRNA products were measured by RT-PCR. A minimum number of three measurements were used to calculate each mean. Graph shows percent exon inclusion as a function of the KCl treatment and differentiation of the cultures. Representative results are shown in the gel panels. The percent change in exon inclusion (ΔEI) was calculated as follows: the percent exon inclusion in the 50 mM KCl sample minus the percent exon inclusion in the 0 mM KCl sample.

Each stage of differentiation of the cultures showed similar trends in which CI cassette exon inclusion decreased (ΔEI, −32% to −36%; Figure 1, lanes 1–12) as a function of the KCl treatment. Based on the consistent response of each exon in this time frame, we used the cultures to ask whether KCl treatment produces transient or stable changes in the splicing patterns of the endogenous RNA transcripts. If transient changes in transcriptional or post-transcriptional events affect splicing factors involved in these mechanisms, we would expect to see a reversal to the basal splicing pattern with time after removal of the stimulus. Reversibility was tested in the 12-d cultures, again using the CI cassette splicing events as a readout. After treatment with KCl, the cultures were changed to fresh medium without KCl, and cells were harvested 0, 6, 12, and 24 h later. KCl-induced changes in the splicing of the CI cassette exon progressively lessened with time to approximately basal levels after KCl washout (ΔEI, −8%; Figure 1, lanes 13–24).

For comparison, we assayed for the effects on alternative exon 9 (E9) of the GABA_A_ receptor γ2 (*GABARG2* gene) in the same cultures, since E9 is known to undergo silencing by the polypyrimidine tract binding protein, but not hnRNP A1 (Figure S1A). Interestingly, E9 inclusion was responsive to the KCl treatment, but this effect was maximized at longer differentiation times of the cultures, and, in contrast to the CI cassette, E9 inclusion increased (ΔEI, 23%, lanes 1–12). These effects were also largely reversible after KCl washout (lanes 13–24). Thus, the KCl-induced effects on CI cassette and E9 inclusion are largely reversible, and suggest a role for mRNA homoeostasis in allowing for the readjustment of splicing patterns to basal levels.

**Figure 2:**
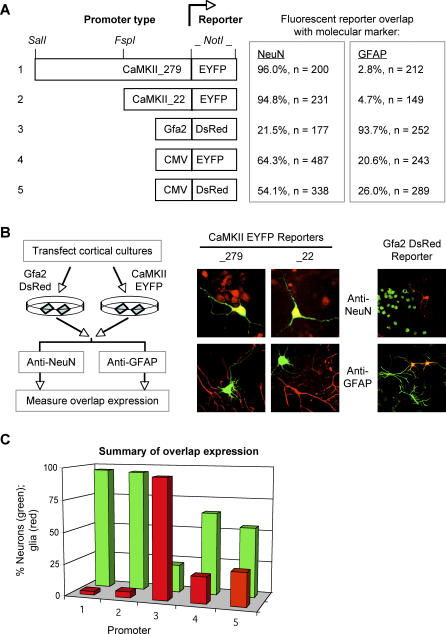
Promoters from Transgenic Mice Drive Neuron- and Glial-Specific Expression in Cortical Cultures (A) Schematic of reporter constructs and results of fluorescent reporter overlap with molecular markers. Full-length (8.5 kb; CaMKII_279), and shortened (2.2 kb; CaMKII_22) mouse alpha CaMKII promoters were fused to the open reading frame of EYFP (constructs 1 and 2). Restriction sites used for the promoter deletions or for insertion of the fluorescent reporter coding sequence are indicated. The glial-specific Gfa2 promoter was fused to DsRed (construct 3). The CMV promoter fused to EYFP (construct 4) or DsRed (construct 5) were used as controls. Numbers at right indicate the percentage of fluorescent cells that showed overlap expression with NeuN or GFAP antibody staining. For each percentage, the total number of fluorescent cells counted for antibody staining *(n)* is indicated. (B) Summary of experimental scheme and representative results. Following transfection, cortical cultures were fixed and stained with antibody for molecular marker as indicated using a FITC or TRITC conjugated secondary antibody (left panel). To measure overlap expression, cells expressing the EYFP (or DsRed) fluorescent reporter were localized, then switched to the channel for detection of TRITC (or FITC) label. Representative results are shown for CaMKII and Gfa2 reporters (right panels). Cells expressing CaMKII EYFP reporters are green; antibody staining is red (TRITC-anti-Neu N, top panels; TRITC-anti-GFAP, bottom panels). Note that the NeuN marker is restricted to the cell nucleus as expected. Cells expressing the Gfa2 DsRed reporter are red; antibody staining is green. Yellow signifies expression overlap. (C) Graphical summary of the fluorescent reporter expression. The *y*-axis represents the percent expression overlap of the fluorescent reporter and antibody staining for neurons (NeuN) or glia (GFAP). The *x*-axis represents promoter constructs from (A).

To experimentally identify nucleotide sequence requirements for activity-induced alternative splicing in neuronal and glial cell types in the cortical cultures, we turned to cell-specific promoters that were previously characterized in transgenic mice. For this purpose, we assessed the ability of the alpha CaMKII promoter to drive neuron-specific expression in rat cortical cultures, since this promoter was shown previously to drive forebrain-specific expression in excitatory neurons of transgenic mice [26]. Fluorescent reporters were constructed in which the expression of enhanced yellow fluorescent protein (EYFP) was driven by various portions of the CaMKII promoter (Figure 2, constructs 1 and 2). Construct CaMKII_279 EYFP contained an 8.5-kilobase (kb) promoter region known to restrict expression of Cre to the forebrain of transgenic mice. For cloning purposes, a shorter promoter region was also tested, which contained the start site–proximal 2.2-kb promoter fragment (CaMKII_22 EYFP).

To determine expression patterns in the cortical cultures, confocal microscopy was used to measure the extent of overlap between EYFP expression and antibody staining with TRITC-labeled NeuN or glial fibrillary associated protein (GFAP), which served as molecular markers for neurons and glia, respectively (Figure 2B). For comparison, we also constructed a fluorescent reporter in which DsRed was fused to a 2-kb region of the Gfa2 promoter, Gfa2 DsRed (Figure 2, construct 3) [27]. The Gfa2 promoter was shown previously to drive expression predominantly in glial cells of transgenic mice [28]. As controls, the EYFP and DsRed coding sequences were also fused to the human cytomegalovirus immediate early (CMV) promoter (Figure 2, constructs 4 and 5).

The strong neuron specificity of expression of the CaMKII promoters in these cultures is shown as the percent overlap expression of each construct with NeuN or GFAP antibody staining (Figure 2A, top right). For each sample, a minimum of 200 cells with EYFP fluorescence were examined for TRITC-labeled NeuN staining. Whereas, the largest (8.5 kb) region of the promoter (CaMKII_279) showed 96% overlap with NeuN and 3% overlap with GFAP, similar results were observed with the 2.2-kb region (CaMKII_22; 95% overlap with NeuN and ∼5% overlap with GFAP). In contrast, the Gfa2 promoter was preferentially expressed in glia in these cultures (94%), and some overlap with neurons was observed (22%) in agreement with previous analysis in transgenic mice [28]. A summary of these results is also shown graphically (Figure 2C). As a reference, EYFP expressed from the CMV promoter (CMV_EYFP) showed 64% overlap with NeuN and 21% overlap with GFAP, whereas the DsRed expressed from the same promoter (CMV_DsRed) showed 54% overlap with NeuN and 26% overlap with GFAP. Due to its mixed expression profile, the CMV promoter was not used for further analysis.

Representative examples of the overlap expression of the two CaMKII EYFP reporters with NeuN is shown in Figure 2B. Overlap expression is indicated by yellow fluorescence in the nuclei of samples stained with TRITC-labeled anti-NeuN (CaMKII EYFP reporters, anti-NeuN panels), and the lack of overlap expression in samples containing the anti-GFAP antibody is clearly shown (anti-GFAP panels). The pattern of expression for samples containing the Gfa2 DsRed reporter characteristically showed overlap of DsRed fluorescence with FITC-labeled anti-GFAP, but not with FITC-labeled anti-NeuN (Gfa2 DsRed panels).

To confirm the neuron- versus glial-specific expression patterns of the promoters, we co-transfected the CaMKII_22_EYFP and Gfa2-DsRed plasmids into the same cortical cultures and determined overlap expression. No greater than 22% overlap would be expected based on the least specific of the two promoters, Gfa2. The observed overlap ranged from 14% to 19% (raw values), in contrast to 82% to 84% from the generally expressed CMV promoters. After normalizing these values to 100% transfection efficiency for the CMV samples, the co-expression of CaMKII and Gfa2 promoters ranged from 17% to 23% (corrected values). These results are in good agreement with the promoter selectivity shown above.

We next asked how these promoters affect splicing patterns in established neuronal (PC12 and ST15A) and muscle myoblast (C2C12) cell lines, in comparison to the primary cortical cultures. Similar CI cassette exon inclusion levels were observed from the two promoter types in the neuronal and non-neuronal cell types (ΔEI, <3.6%, Table 1). Thus, the increase in CI cassette exon inclusion arising from the promoter fusions in primary cortical cultures must reflect the properties of the neurons and glial cells in the cultures, and cannot be explained by the promoters alone.

**Table 1:**
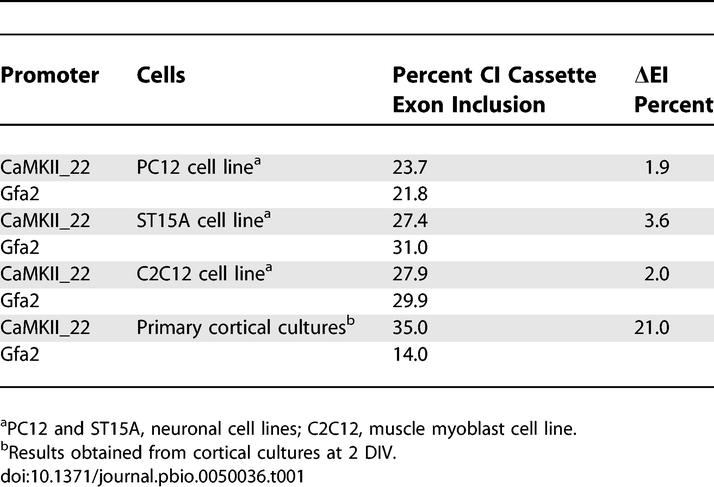
Effects of Cell-Specific Promoters on Alternative Exon Inclusion in Cell Lines versus Cortical Cultures for the Splicing Reporter CI wt0

**Figure 3:**
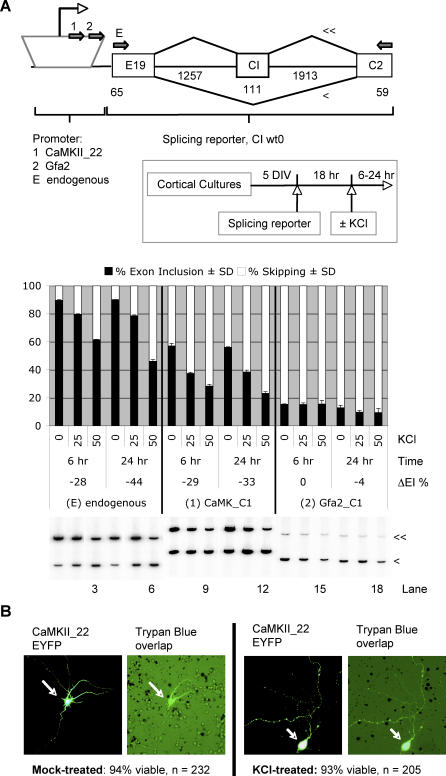
Cell-Specific Splicing Reporter Assay for Depolarization Induced Alternative Splicing and Effects on Cortical Neuron Viability (A) Changes in C1 cassette exon inclusion in response to depolarization were measured by transfecting CaMKII_22 and Gfa2 C1 wt0 splicing reporters into day 5 (DIV) cortical cultures, followed by addition of KCl (25 mM or 50 mM) into the culture medium for 6 or 24 h to induce depolarization. Inset summarizes order of addition. Control cultures were mock treated in parallel (0 mM KCl). Splicing patterns were determined from RNA harvested from the samples by RT-PCR using forward primers (rightward arrows) specific for transcripts expressed from CaMKII_22 (1) and Gfa2 (2), together with a common reverse primer specific for the C2 exon (leftward arrow). Endogenous transcripts were amplified similarly with a forward primer (E) together with the C2-specific primer. A minimum number of three measurements were used for each mean and standard deviation shown. Structures of the splicing reporters are shown; exon numbers are based on the human GRIN1–006 transcript (schematic, top). Bar graphs represent percent C1 exon inclusion at two time points (6 and 24 h) after addition of KCl. Representative gels are shown (bottom). Effects on splicing reporters, (1) CaMKII_22 (lanes 7–12) and (2) Gfa2 (lanes 13–18), are shown with those of the corresponding endogenous (E) *GRIN1* mRNA (lanes 1–6). Induced changes in splicing (ΔEI, %) were calculated as described above (Figure 1). The exon-included (<<) and -skipped (<) mRNAs are indicated. SD, standard deviation. (B) Viability of CaMK-expressing cortical neurons following KCl-mediated depolarization. Cortical neurons were transfected with CaMKII_22 EYFP plasmid, and treated under mock or depolarization conditions (0 and 50 mM KCl, respectively) as described for (A). Cultures were stained with trypan blue and more than 200 EYFP-expressing neurons were scored for trypan blue staining. The percentage of viable cells for each condition is shown; *n* = total cells scored. Representative neurons from these cultures are shown. Left panels of each condition show CaMKII-EYFP fluorescence. Right panels show bright field (trypan blue) and fluorescence together.

Next we took advantage of the CaMKII and Gfa2 splicing reporters to characterize the KCl-induced effects on CI cassette exon silencing as a function of cell type in the primary cortical cultures. Wild-type CI cassette splicing reporters driven by the cell-specific promoters CaMKII_22 CI wt0 and Gfa2 C1 wt0 were transiently expressed in cortical cultures for 18 h, followed by treatment with KCl for 6 and 24 h. When transcripts were expressed from the CaMKII_22 promoter, CI cassette exon inclusion decreased in response to the KCl treatment, and this effect was consistent in neurons at all time points examined (Figure 3A, lanes 7–12). Similar effects were observed for the endogenous CI cassette exon when mock-transfected cultures were treated in parallel (lanes 1–6). Optimal effects were reached after 24 h for both endogenous and CaMKII_22-expressed transcripts. In contrast, when transcripts were expressed from the Gfa2 promoter, CI inclusion showed little or no change at the 6- and 24-h time points (lanes 13–18). Based on the cell specificity of the CaMKII promoter shown above, we conclude that neurons play the predominant role in the KCl-induced changes in alternative splicing in these cultures. Furthermore, the trend and magnitude of the effects for the exogenous CI cassette exon inclusion are in good agreement with those of the endogenous transcripts. As a corollary to this experiment, we measured the number of CaMKII_EYFP-expressing neurons that fail to exclude trypan blue. In mock- and depolarization-treated cultures, we observed that the viability of the neurons was high in both the mock-treated (94% viable) and depolarization-treated (93% viable) samples (Figure 3B). Thus, we conclude that the RT-PCR results shown above (Figure 3A, lanes 7–12) largely reflect splicing pattern changes in viable neurons.

To further validate these results, we transferred the E9 splicing reporter into the CaMKII and Gfa2 promoter constructs and tested their response to KCl treatment in the cortical cultures (Figure S1B). When expression was driven in neurons, E9 inclusion increased similarly to that of the endogenous transcripts (ΔEI, 17% and 15%, respectively), but this was not the case when expression was driven in glial cells (ΔEI, 3%). Thus, for a second test exon, the promoter system recapitulated the splicing response in neurons as observed for the endogenous transcripts.

### Identification of RNA Sequence Motifs That Mediate the Response of Exon Skipping to Neuronal Activity

**Figure 4:**
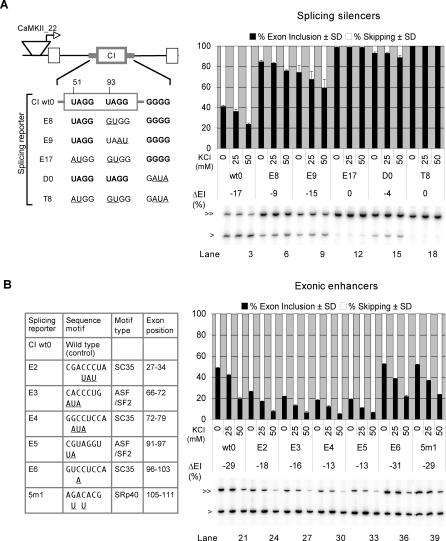
Nucleotide Sequence Requirements for KCl-Induced Depolarization Effects on Splicing in Primary Neurons (A) Splicing silencer requirement for the induced response. Splicing reporters based on CI wt0 were expressed in primary neurons via the CaMKII_22 promoter, and depolarization was induced by addition of KCl as in the experiment of Figure 3. Structures of splicing reporters are identical except for point mutations in silencing motifs as shown in the schematic. UAGG silencing motifs at positions 51 and 93 of the CI cassette exon and a GGGG motif in the downstream intron are shown for the wild-type substrate, CI wt0. Mutations in splicing reporters, E8, E9, E17, D0, and T8, are underscored. All splicing reporters [25] were subcloned and expressed from the CaMKII_22 promoter. Graph displays effects of depolarization on the splicing of the wild-type and mutant substrates. RT-PCR analysis was performed with forward primer (1) and the common reverse primer as for Figure 3. A representative gel panel is shown. Exon-included (>>) and exon-skipped (>) mRNAs are indicated. ΔEI values are shown. (B) Effects of exonic splicing enhancers. Point mutations in six exonic enhancers of the CI cassette exon were tested as described in (A). Sequence and position of SC35, ASF/SF2, and SRp40 motifs are shown at left; mutations within each motif are underscored. Effects of mutations as a function of depolarization in neurons and ΔEI values are shown. SD, standard deviation.

Based on the well-behaved exon-skipping response of the CI cassette exon to KCl-induced depolarization in this system, we focused on this exon to identify nucleotide sequences involved in the response. A three-component code of two exonic UAGGs and an intronic GGGG motif, which was recently shown to mediate CI cassette exon silencing [29], served as the starting point for these experiments. Splicing reporters with point mutations in combinations of the three silencing motifs were subcloned under the control of the CaMKII_22 promoter, and assayed in the cortical cultures (Figure 4A).

In the absence of KCl treatment, the CI wt0 reporter with intact UAGG and GGGG motifs showed strong silencing activity (exon skipping) in the primary neurons in agreement with our previous analysis in cultured cell lines. Compared to the wild-type substrate, point mutations in a single silencer increased exon inclusion (Figure 4A, lanes 1, 4, 7, and 13), and multiple point mutations generated complete or nearly complete exon inclusion (lanes 10 and 16). In results new to this study, the UAGG and GGGG silencer motifs were found to be important for the depolarization-induced effects on exon skipping in the primary neurons. A complete disruption of KCl-induced exon skipping was associated with point mutations in two or more of the silencing motifs (substrates E17 and T8; lanes 10–12 and 16–18). In contrast, an increase in exon skipping occurred in parallel with the depolarization treatment for the wild-type substrate (ΔEI, −17%; lanes 1–3). Point mutations in single motifs showed a similar trend in which the KCl-induced effects on exon skipping were disrupted at various levels (substrates E8, E9, and D0; lanes 4–9 and 13–15). We next tested the role of exonic enhancer motifs in the same fashion, since these motifs function generally to antagonize exon silencing. The sequence, type, and position of the exonic enhancer motifs and their inactivating mutations are shown in Figure 4B. Of the six mutants, E2, E3, E4, and E5 showed reduced basal levels of CI exon inclusion in neurons, and the response of these exons to KCl-induced depolarization was either similar to (E6 and 5 m1, lanes 34–39) or reduced (E2, E3, E4, and E5, lanes 22–33) compared to the wild-type CI cassette (lanes 19–21). These results suggest that sequences outside of the UAGG motifs in the exon may also play a role in the induced splicing silencing response.

**Figure 5:**
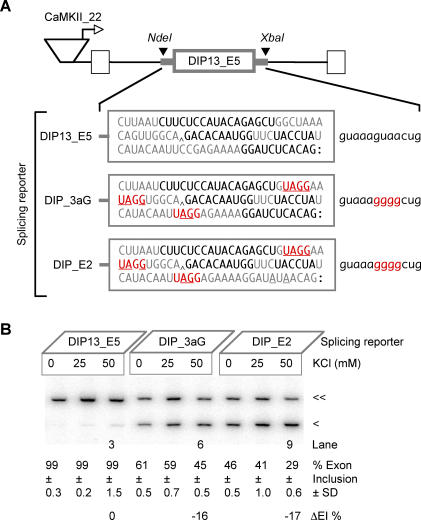
Transfer of Multicomponent UAGG Silencing Motif Pattern Confers Sensitivity of a Constitutive Exon to Depolarization in Primary Neurons (A) The schematic at top illustrates the heterologous splicing reporters used for the transfer experiments. Exon 5 of the DIP13 transcript and 12 nucleotides of each flanking intron was inserted between the NdeI and XbaI restriction sites of the SIRT1a_CaMKII22 plasmid. An engineered EcoR1 site is indicated by the arrowhead in the sequences shown. Intron/exon lengths (nucleotides) are as follows: exon 1, 308; intron 1, 340; exon 2, 94; intron 2, 287; and exon 3, 436. For the middle exon sequences shown in the expanded region for wild-type exon 5, the predicted exonic enhancer motifs are highlighted in black letters. UAGG silencing motifs were introduced into the middle exon of the DIP_3aG, and DIP_E2 splicing reporters as indicated (red letters); mutations are underscored. Colon indicates 5′ splice site cleavage position. The first 12 nucleotides of the downstream intron are shown at right for the wild-type substrate (DIP13_E5). The GGGG motif at intron nucleotides 6–9 is highlighted in red. (B) Representative results of RT-PCR analysis and values for percent exon inclusion and **Δ**EI are shown; the exon-included (<<) and -skipped (<) mRNAs are indicated. SD, standard deviation.

To validate these results, we asked whether a multicomponent UAGG silencing motif code is sufficient to confer sensitivity to KCl-induced depolarization; we introduced UAGG motifs into exon 5 of the gene encoding the human Dip13 beta adapter protein (DIP13B_HUMAN). This exon was chosen based on the use of algorithms that predicted strong 5′ and 3′ splice sites, a moderate number of exonic enhancer motifs (*n* = 10), and a lack of known exonic silencing motifs (MaxEntScan, ACEScan, and ESEFinder). Another attractive feature was the clustered arrangement of predicted enhancer motifs that allowed for the insertion of UAGG motifs at multiple discrete positions. The region containing exon 5 and its flanking splice sites was cloned into a chimeric splicing reporter by replacing the middle exon of the previously described SIRT1a plasmid [29], and the promoter was replaced with CaMKII_22 to direct expression in neurons (Figure 5A, DIP13_E5). When expressed in neurons, the DIP13_E5 splicing reporter was nearly insensitive to the excitation treatment (ΔEI, 0%; Figure 5B, lanes 1–3). In order to test the role of the silencing motif pattern in the response to excitation, we next introduced three exonic UAGG motifs and a 5′ splice site GGGG motif (DIP_3aG). Introduction of the silencing motif pattern indeed generated an exon-skipping pattern in resting neurons (61% exon inclusion), and in the presence of KCl treatment to induce excitation, exon inclusion progressively decreased to 45% (ΔEI, −16%; lanes 4–6). In another variant, DIP_E2, point mutations were introduced into the exon to destroy overlapping SC35 and ASF-SF2 motifs near the 3′ end of the exon (Figure 5A). For DIP_E2, exon inclusion decreased in resting neurons, and the response to excitation was similar to that observed for DIP_3aG (Figure 5B, lanes 7–9). Thus, we conclude that a multicomponent UAGG silencing motif code is sufficient to generate induced exon skipping in response to KCl-induced depolarization.

### Biochemical Analysis of Protein Binding to Silencing Motifs in Nuclear Extracts from Resting and Excited Cultures

We reasoned that factors known to be involved in splicing silencing via UAGG silencing motifs in resting cells would be likely candidates to mediate the induced splicing silencing response in excited neurons. Alternatively, excitation might weaken the role of factors involved in antagonizing this silencing mechanism. We initially focused on hnRNP A1 to probe for changes in regulatory factors, because our previous work demonstrated direct binding of this factor to the UAGG motifs in HeLa nuclear extracts, and because its silencing effect in vivo was dependent upon the intact motif pattern.

To attempt to visualize whether changes in hnRNP A1 accompany KCl-induced depolarization, RNA-protein binding assays were used as sensitive readouts. In these experiments, 5-d cortical cultures were treated with KCl in the medium to induce depolarization, and cells were harvested for preparation of small-scale nuclear extracts (see Materials and Methods). The time of KCl treatment was shortened from 24 h to 12 h based on the expectation that any excitation-induced alterations to splicing factors should precede the splicing pattern shifts themselves.

**Figure 6:**
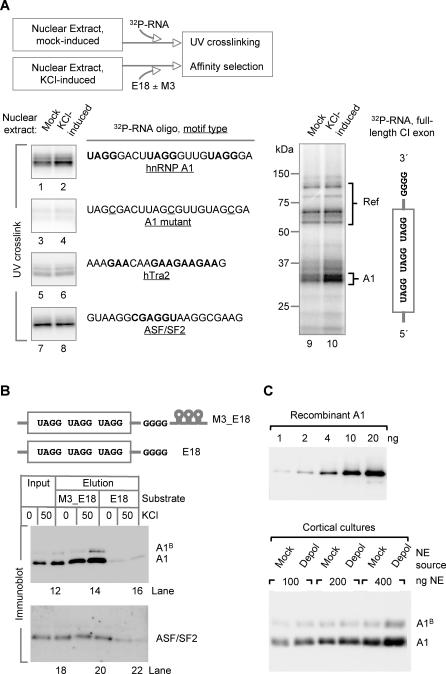
Biochemical Analysis of Protein Binding to UAGG Silencer Motifs as a Function of Cell Excitation (A) Schematic of methodology used to analyze protein binding to RNA substrates in nuclear extracts from KCl-induced and mock-treated cultures. UV crosslinking assays were performed under splicing conditions to monitor protein binding to radiolabeled RNA oligos (lanes 1–8), and to the full-length CI cassette exon (lanes 9 and 10). Sequences of RNA oligos and schematic of CI exon are shown at right of gel panels. Gel panels show radiolabeled proteins after SDS-PAGE separation; kDa ladder indicates molecular weight standards. (B) Affinity selection of proteins non-covalently bound to RNA substrates containing the full-length CI cassette exon with (M3_E18) or without (E18) the M3 hairpin. Assembly reactions containing RNA substrates pre-bound to MS2-MBP were incubated under splicing conditions, bound to amylose columns, and eluted with maltose. Samples were resolved by SDS-PAGE and transferred to nylon membranes for immunoblotting with hnRNP A1-specific 9H10 (lanes 11–16) or ASF/SF2 (lanes 17–22) antibody. A1 and A1^B^ represent the major and minor isoforms of hnRNP A1, respectively. (C) Quantitative Western blots show serial dilutions of recombinant hnRNP A1 (MBP-A1) grown in Escherichia coli as reference standards from 1 to 20 ng (top panel). Nuclear levels of hnRNP A1 and A1B are shown at three loading levels, 100, 200, and 400 ng of total nuclear extract (NE) protein (bottom panel). Nuclear extracts were prepared from mock-treated (Mock) and 50 mM KCl-treated cultures (Depol). Monoclonal antibody 9H10 was used to detect hnRNP A1 in both blots.

Ultraviolet (UV) crosslinking was used to monitor direct protein binding to 5′[^32^P]-labeled RNA oligonucleotides under splicing conditions, and the crosslinked proteins were resolved by SDS-PAGE. A 22-mer containing three UAGG motifs showed increased crosslinking of a 34-kDa protein, suggestive of hnRNP A1, in nuclear extracts prepared from KCl-induced versus mock-treated samples (Figure 6A, lanes 1 and 2). Increased crosslinking of the 34-kDa protein in the KCl-induced extracts was observed in eight different nuclear extract preparations, and the fold increase was in the range of 1.5- to 2.5-fold. To determine whether hnRNP A1 was responsible for the observed increase in crosslinking, a size-matched control RNA with point mutations in the UAGG motifs (A1 mutant) was tested in parallel reactions. Crosslinking of the 34-kDa protein to the A1 mutant oligo was abolished, demonstrating that intact UAGGs are essential for binding. To confirm the identity of the 34-kDa protein, three UV crosslinking reactions equivalent to that shown in lane 2 were combined and immunoprecipitated with monoclonal antibody 9H10, which is specific for hnRNP A1. These results showed that, relative to the control antibody, the 34-kDa crosslinked protein was partitioned into the pellet (and depleted from the supernatant) in the presence of 9H10, supporting its identification as A1 (Figure S2). For comparison, parallel crosslinking reactions with distinct RNA oligos specific for human Tra2 and ASF/SF2 were also tested, but these proteins showed no detectable change in binding in extracts prepared from the KCl-induced versus mock-treated cultures.

Enhanced hnRNP A1 binding was also observed for the full-length CI cassette exon in UV crosslinking reactions prepared with nuclear extracts from KCl-induced cultures (Figure 6A. lanes 9 and 10). Note that the full-length CI cassette exon tested here contains three UAGGs distributed throughout the 111-nucleotide exon, which is a less-concentrated motif arrangement relative to the A1 oligo. In contrast to hnRNP A1, higher molecular weight proteins in the 57- to 100-kDa range in these samples serve as reference comparisons, since these displayed similar crosslinking levels in the two nuclear extracts (lanes 9 and 10, Ref bands). The identity of hnRNP A1 was verified by competition experiments with unlabeled A1 oligo, which reduced crosslinking to the 34-kDa protein in a concentration-dependent manner (unpublished data).

To verify the change in hnRNP A1 binding observed in the UV crosslinking assay, affinity selection was used as a complementary method. For this experiment, the full-length CI cassette exon with three UAGGs was subcloned upstream of the M3 hairpin to provide an affinity tag that was specific for the MS2-MBP fusion protein [30]. The CI cassette exon was subcloned without the M3 hairpin as a control. To assay for hnRNP A1, one half of the eluted samples were separated by SDS-PAGE and immunoblotted with the 9H10 antibody. Parallel blots of the second half of the samples were developed with an antibody specific for ASF/SF2. Binding of hnRNP A1 was dependent upon the presence of the M3 hairpin (Figure 6B, lanes 13–16), and an increase (1.5-fold) in A1 binding to the M3_E18 substrate was observed in the KCl-induced versus mock-treated extracts (lanes 13 and 14). This difference in A1 was similar in the input samples prior to RNA binding (lanes 11 and 12). The increase in the level of A1 protein was similar to the input samples prior to RNA binding. In contrast, ASF/SF2 showed no change in binding to the M3_E18 substrate in the two samples, nor was there an observable difference in ASF/SF2 in the input samples (lanes 17–22).

To confirm the difference in nuclear levels of hnRNP A1, quantitative Western blotting was used to measure A1 levels in nuclear extracts from resting and excited cultures relative to known levels of recombinant A1 (Figure 6C). These results verify that an increase in nuclear A1 protein levels in the cultures is associated with the depolarization treatment.

### Evidence for the Role of the NMDA Receptor and Calcium Signaling in the Induced Splicing Response

**Figure 7:**
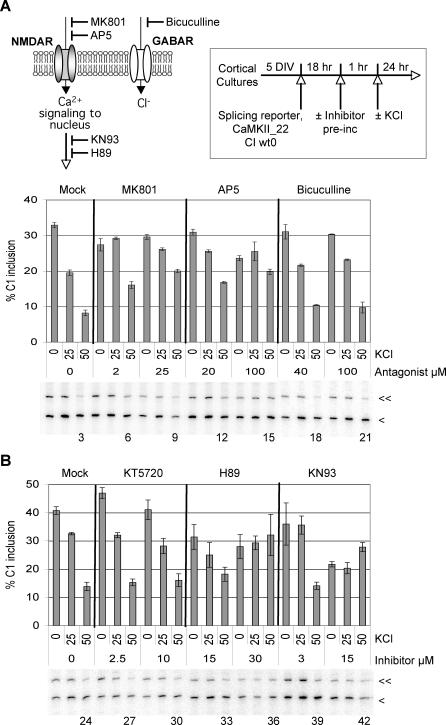
Test of Inhibitors of NMDA Receptors and Signaling Pathways on the CI Cassette Exon-Skipping Response in Excited Neurons (A) Schematic summarizes the experimental procedure: cortical cultures were transfected with CaMKII_22 CI wt0, and pre-incubated with inhibitor at final concentrations as indicated just above gel panels. With the inhibitor remaining in the culture medium, KCl was then added (25 or 50 mM) to induce depolarization. Gel panels and graph in (A) show effects of NMDA receptor antagonists MK801 and AP5. Pre-inc, pre-incubated. (B) Gel panels and graph shows the effects of cell-permeable inhibitors, KT5720, H89, and KN93. Experiments were performed as described for (A). The exon-included (<<) and -skipped (<) mRNAs are indicated.

We would expect cell excitation initiating at the cell membrane to communicate changes to splicing factors in the nucleus via calcium-mediated signal transduction pathways. In the absence of antagonists, the NMDA receptor channel opens in response to membrane depolarization and serves as the major conduit for calcium entry into neurons. Because NMDA receptors are functionally intact in neurons in these cultures, we wished to take advantage of this property of the culture system to ask whether NMDA receptors play a role in the induced effects on splicing observed here. To address this question, we expressed the CI wt0 splicing reporter in 5-d cortical cultures under the control of the CaMKII_22 promoter, and the cultures were pre-incubated with antagonists specific for the NMDA receptor before KCl-induced depolarization. MK801 and AP5 antagonists were used for these experiments because of their known specificity and effectiveness in inhibiting the NMDA receptor calcium channel in cortical cultures [31,32]. AP5 binds competitively to the extracellular glutamate (NMDA) site of the NR2 subunit, whereas MK801 binds to the channel itself, blocking ion flow. The exon-skipping response of the CI cassette exon was strong in the control (mock treated) samples (Figure 7A, lanes 1–3), but in the presence of either antagonist, this response was attenuated in a dose-dependent manner (lanes 4–15). Relative to the control sample (ΔEI, −25%), exon skipping decreased in the presence of MK801 (ΔEI, −10%) and in the presence of AP5 (ΔEI, −4%). In contrast, when cultures were pre-incubated with bicuculline, which is an antagonist of the GABA_A_ receptor, only slight effects were observed on induced exon skipping (ΔEI, −22%; lanes 16–21) relative to the control (ΔEI, −25%; lanes 1–3). Thus, to a first approximation, the effects of MK801 and AP5 implicate a role for the NMDA receptor in mediating the exon-skipping response of the CI cassette exon in this system.

To extend these results, we used cell-permeable inhibitors to ask whether calcium-mediated signal transduction pathways downstream of NMDA receptors are involved in the induced splicing silencing response of the CI cassette exon. Relative to the mock-treated control, the exon-skipping response was strongly inhibited in the presence of 15 μM KN93, which is an active site-based inhibitor of CaMK I, II, and IV (Figure 7B, lanes 22–24 and 40–42), and this effect was lessened when the inhibitor concentration was reduced to 3 μM (lanes 37–39). The control compound, KN92, had little or no effect in these experiments (P. An and P. J. Grabowski, unpublished data). We also found that the exon-skipping response was inhibited by H89, which has been widely used as an inhibitor of PKA (lanes 31–36). In an attempt to confirm this effect, the inhibitor KT5720, which is reported to have a higher specificity for PKA, was also tested. Interestingly, KT5720 had little or no effect at 2.5 and 10 μM concentrations (lanes 25–30), indicating that the inhibitory effect of H89 on splicing silencing observed here may involve a distinct pathway (or pathways).

To determine if additional calcium channels could play a role in the splicing response of the CI cassette exon, we tested the effects of specific antagonists of the AMPA/kainate receptor and voltage-gated calcium channels (L- and N-type) (Figure S3). Relative to control samples, the exon-skipping response was reduced, but not eliminated, in the presence of nimodipine (antagonist of L-type calcium channels) and conotoxin (N-type calcium channels), whereas CNQX (AMPA/kainate receptors) showed little or no effect. Taken together, these results are consistent with roles for multiple calcium channels in the induced splicing response of the CI cassette exon in neurons.

### Depolarization Alters Splicing Patterns of Additional Transcripts with Synaptic Functions

**Figure 8:**
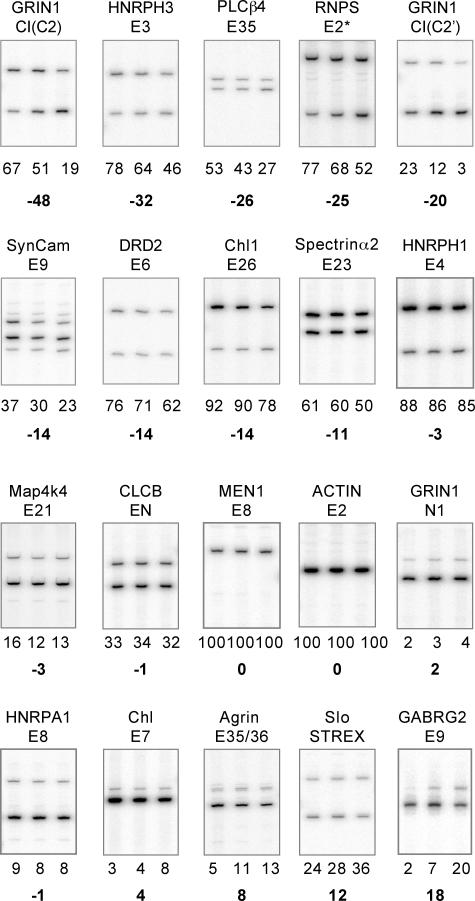
Exon-Skipping Responsiveness of Fourteen Endogenous Exons in Cortical Cultures During Depolarization Treatment Gel panels represent RT-PCR analysis of endogenous exons with gene and exon numbers as indicated above each panel. Each panel represents 24-h treatment of cortical cultures with 0, 25, and 50 mM KCl (lanes, left to right). One PCR primer in each sample was 5′ end labeled with ^32^P. Raw exon inclusion values (EI) and change in exon inclusion values (ΔEI; boldface) are given below each panel. E8 of MEN1 and E2 of actin are constitutive exons; all other exons are alternative cassette exons. A single asterisk (*) indicates 6-h KCl treatment.

Next, we expanded the analysis of endogenous transcripts in the cortical cultures to test whether additional alternative cassette exons were responsive to depolarization, and if so, to determine whether exonic UAGG silencing motifs were required. We were also prompted to examine transcripts that encode protein components with synaptic functions, since the NMDA NR1 receptor and other calcium channels were implicated in the splicing response of the CI cassette exon from the experiments described above. Of 14 new exons tested, seven showed a significant exon-skipping response, five showed little or no response, and two showed an increase in exon inclusion (Figure 8).

**Figure 9:**
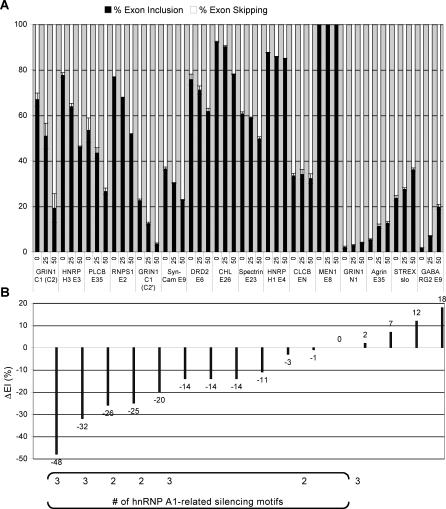
Summary of Exon-Skipping Responsiveness and UAGG Code (A) Percent exon inclusion values are shown graphically for the samples represented in Figure 8. (B) Percent ΔEI values represent exon-skipping responses to depolarization for samples in Figure 8. Negative values (≤−10%) reflect significant exon skipping, whereas positive values (≥10%) reflect exon inclusion. The number of UAGG (hnRNP A1-type) silencing motifs are indicated (bottom).

Strong exon-skipping responders were found to contain hnRNP A1–type silencing motifs in or near the responsive exon (Figure 9). Exon 3 of hnRNP H3 and exon 2 of the RNPS1 transcripts (ΔEI, −32% and −25%, respectively) contain either one exonic UAGG and a 5′ splice site GGGG motif (H3), or two exonic UAGGs (RNPS1). Exon 35 of PLCβ4 also showed a strong response (ΔEI, −26%), and this exon contains two TAGA motifs, which reflects a four of six match to hnRNP A1 motifs reported for SMN2 exon 7 and human CD44 v5 exon [33]. These motifs cannot be the sole determinants of the response, however. Exon 4 of hnRNP H1, which is identical in size (139 nucleotides) and highly homologous to exon 3 of hnRNP H3, showed little or no response (ΔEI, −3%) in the same samples. Interestingly, there is one exonic UAGG and a GGGG tetramer near the 5′ splice site of this exon, but it lacks a third silencing motif (exonic GGGG) that is found in exon 3 of hnRNP H3. Moreover, constitutive exon 8 of the MEN1 transcript remains entirely unresponsive even though this exon contains two exonic UAGGs and a 5′ splice site proximal GGGG motif.

Of the exons that showed little or no response, exon EN of *clathrin light chain B (CLCB)* was the most interesting. In a previous report, this exon showed increased exon skipping in the cortex and hippocampus of rats treated with pilocarpine [13]. In cortical cultures under the conditions tested here, however, exon EN showed a stable splicing pattern even though it is a well-skipped exon (exon inclusion, 30%). Other alternative exons with a predominant skipping pattern that were unresponsive in these cultures include exon 21 of MAP4k4, exon 8 of hnRNP A1, exon N1 of *GRIN1,* exon 7 of *Chl,* and exon 7 of *Agrin*. These exons, including EN of *CLCB,* lack hnRNP A1 motifs. Thus, the exon-skipping responsiveness of certain exons to depolarization treatment cannot be explained solely by a weakening of the general splicing machinery that causes all skipped exons to undergo stronger exon-skipping responses.

Finally, two exons showed moderate increases in exon inclusion during the depolarization treatment. These included exon 9 of the GABA_A_ receptor γ2 transcript, and the STREX exon of the c-*slo* transcript (ΔEI, 18%, and 12%, respectively). These results suggest that some of the biochemical changes resulting from the depolarization treatment in neuronal cells can, in principle, increase exon inclusion.

## Discussion

### UAGG-Type Silencing Motifs Mediate Exon-Skipping Responsiveness to Neuronal Excitation

At the level of pre-mRNA splicing, the CI cassette of the NMDA R1 receptor is one of several responsive exons that undergo an increase in exon skipping in response to neuronal excitation. Despite the importance of NMDA receptors in neuronal excitability at the level of ion channel function, the mechanisms by which stimuli are communicated to the nuclear splicing machinery to affect changes in alternative splicing are poorly understood. Moreover, methodologies to address these questions in primary neurons, in which NMDA receptors are functionally intact, have not been well developed.

Here we describe a promoter-based expression assay that reports splicing changes in response to cell excitation with tight specificity for the neurons of cortical cultures derived from embryonic rat brain. Due to its neuron-specific expression in the forebrain regions of transgenic mice, the alpha CaMKII promoter was tested for its ability to provide a neuron-specific readout in a primary culture setting. In cortical cultures, full-length as well as truncated forms of this promoter displayed a strong preference for expression in neurons versus glial cells as demonstrated by co-localization of promoter-directed EYFP expression together with antibody staining for cell-specific markers. Moreover, these effects were dependent upon the primary culture context, since neuron-specific splicing patterns were eliminated when the same promoter-EYFP cassettes were expressed in neuronal versus non-neuronal cell lines.

Using splicing reporters driven by the CaMKII promoter, we show that neuronal cells play the predominant role in the acute splicing response to excitation, and these splicing pattern changes reflect the trend and magnitude of those observed for the corresponding endogenous transcripts. In neurons of these primary cultures, the exon-skipping response of the CI cassette exon was mediated by combinations of UAGG-type silencing motifs. The general importance of a strong silencing motif pattern was confirmed by a gain-of-responsiveness due to the transfer of a similar UAGG-type silencing motif pattern into a constitutive exon. In this heterologous context, the transfer of multiple exonic UAGG motifs and a 5′ splice site proximal GGGG motif generated a basal-level exon-skipping pattern in the absence of depolarization, and exon skipping was further increased, over and above this basal level, as a function of depolarization. Furthermore, by expanding this analysis to fourteen alternatively spliced exons, several exons with strong exon-skipping responses were found to contain multiple hnRNP A1 silencing motifs consistent with a plausible role for hnRNP A1 in these effects. These results extend our previous work, which identified a multicomponent UAGG and GGGG motif code for basal-level splicing silencing of the CI cassette exon in established cell lines [29]. Additional studies have documented a similar exon-skipping response of the CI cassette exon to cell excitation in rat brain [13] and in GH3 pituitary cells, but in these previous studies nucleotide sequences required for the effects were not defined.

The first RNA element shown to confer an exon-skipping response to KCl-mediated depolarization was defined for the STREX exon of the BK potassium channel. This element, termed CaRRE (CaMKIV-responsive RNA element), was defined as a 54-nucleotide pyrimidine-rich region in the 3′ splice site of the STREX exon, and a pyrimidine-rich exonic RNA element, termed D56, was also found to contribute to this splicing response in GH3 cells [12]. The CaRRE sequence was later refined by mutagenesis in primary mouse cerebellar cultures to comprise the sequence (5′) CACATNRTTAT (3′) [34]. We note that neither the CaRRE, nor the D56 element, are present in the 3′ splice site upstream of the CI cassette. Moreover, the UAGG-type silencing motifs defined here for the responsiveness of the CI cassette are absent from the STREX exon. These observations suggest that the exon-skipping responsiveness of these two exons is likely to involve distinct silencing mechanisms. The accompanying paper by Lee et al.[35] reports the identification of two types of CaRRE elements required for inducible exon-skipping in P19 cells, one of which is adjacent to a UAGG splicing silencer in the CI cassette. Whether the sequence requirements for inducible exon-skipping are more complex than these studies indicate or whether the differences in cell types or assays can account for seemingly different requirements is unknown.

In efforts to extend the results of the RNA motif analysis, we used biochemical approaches to attempt to visualize any changes in the proteins that bind directly to the identified UAGG motifs. In UV crosslinking experiments, the binding of hnRNP A1 to UAGG motifs was found to increase in nuclear extracts from the excited versus resting cells (2-fold increase), and this was verified by affinity selection and immunoblotting. In contrast, no change in the binding of splicing factors ASF/SF2 and TRA2 to their respective RNA substrates was observed in parallel reactions using these nuclear extracts. In addition, the cell-permeable PKA inhibitor H89, which was shown to block the excitation effects at the level of splicing, was also found to eliminate the increase in hnRNP A1 crosslinking in nuclear extracts that were excited in the presence of this compound. Taken together with the UAGG motif analysis, these results are in agreement with the prediction that signal transduction pathways communicate with splicing regulatory factors to cause induced effects on splicing, although at this point, many questions remain about how this communication is relayed to the nucleus, and exactly how the hnRNP A1 polypeptide is affected by the induction. Previous studies have documented an increase in the cytoplasmic distribution of A1 together with specific changes in the phosphorylation pattern upon osmotic stress [19,20]. Despite the apparent differences in the nature of the changes in hnRNP A1 during osmotic stress versus neuronal excitation, these previous studies and the results shown here, reinforce the idea that A1 is adept at responding to environmental signals.

### Implications for the Calcium Channel of the NMDA Receptor in Splicing Responsiveness to Neuronal Excitation

NMDA receptors are multisubunit protein complexes concentrated in the postsynaptic membrane where they regulate calcium influx into neurons in response to the binding of glutamate and glycine. Calcium influx through NMDA receptors is believed to trigger acute biochemical changes leading to long-term changes in synaptic strength, or plasticity [36]. In this study, we have taken advantage of the cortical culture system to explore the potential role of NMDA receptors in the CI cassette exon-skipping response to neuronal excitation. If the excitation regimen used here to induce the CI cassette exon-skipping response involves calcium influx through NMDA receptors, we would expect antagonists that specifically block the NMDA calcium channel to weaken the excitation effects on splicing. Consistent with this idea, we observed a substantial, dose-dependent block in the response of this exon-skipping event when the cultures were treated with the antagonists AP5 or MK801. In contrast, when bicuculline was applied as an antagonist of the GABA_A_ receptor, the exon-skipping response was only slightly weakened. The complete inhibition of the CI cassette exon-skipping response by KN93 is consistent with a role for calcium-mediated signaling in these effects. The effects shown here for KN93 in cortical neurons are in good agreement with previous studies from the Black laboratory, which have demonstrated a role, specifically, for CaMKIV in the exon-skipping response to excitation in GH3 cells and in cerebellum tissue from mouse CaMKIV knockout lines [12,34]. Because treatment with AP5 or MK801 largely, but not completely, blocks the CI cassette exon-skipping response to excitation, it is a viable possibility that calcium influx into neurons via AMPA receptors and/or L-type calcium channels might also be involved.

Modular changes in a variety of proteins involved in synaptic function have been documented at the level of alternative splicing. A large-scale analysis of splicing pattern changes in the neocortex of Nova2 knockout mice has revealed that approximately 34 exons with splicing defects correspond to transcripts that encode synapse-related proteins [37,38]. Of these, the CI cassette exon was found to undergo increased exon skipping (∼45% increase) in the Nova2 knockout mice, suggesting a role for Nova2 as a positive regulator of this exon in the mouse forebrain. In addition, NAPOR/CUGBP2, hnRNP H, and to a lesser extent ASF/SF2 and SC35, have been shown to function as positive regulators of the CI cassette exon at the level of splicing [29,39]. Along with the splicing silencing features of this control mechanism, a significant amount of combinatorial control of the CI cassette exon is indicated. Although it is not known how the nuclear levels of these additional factors are affected by neuronal excitation, an assumption of the present study is that selective alteration(s) to the balance of enhancement versus silencing underlies the net changes observed in the splicing patterns.

To a first approximation, our results indicate that NMDA receptors are involved in the CI cassette exon-skipping response to KCl-induced depolarization in these cultures. This raises questions about the potential biological role of this exon-skipping response. The CI cassette exon itself has been shown to be important for localization of the NMDA R1 subunit of the receptor at the plasma membrane, and the role of this exon in intracellular signaling has been documented [40,41]. Previous studies have shown that NMDA receptor recruitment to synaptic sites can be substantially affected by neuronal activity. Rao and Craig found increased accumulation of NMDA receptors at synaptic sites in hippocampal cultures when neuronal excitation was chronically blocked by treatment with AP5, although effects at the level of splicing were not detected [42]. In another study, the Ehlers laboratory showed that trafficking of NMDA receptors to synapses during activity blockade in cortical cultures was associated with spliced variants of the NMDA R1 receptor that contain the C2′ exon, but not the alternative exon, C2 [43].

The synaptic homeostasis model holds that, in order to stabilize excitability, neurons tend to downwardly adjust their synaptic strengths under conditions of chronic excitation, or upwardly adjust them under conditions of activity blockade [44]. This model, together with the results shown here, suggests that alternative splicing of the CI cassette exon might be involved in strategies by which neurons mount protective responses to an overload or lack of environmental stimuli. In agreement with the results shown here, a chronic KCl-induced depolarization of cortical cultures was shown to accompany a decrease in the NR1–1 (+CI cassette), and an increase in the NR1–2 (−CI cassette), mRNA isoform of the NMDA R1 receptor [45]. We have no direct evidence that a 2-fold increase in exon skipping of the CI cassette exon would cause a meaningful change in the number of NMDA receptors at the synapse. Nonetheless, it is apparent from other studies that subtle changes at the level of splicing can bring about important changes at the level of protein expression. In mice, a splicing defect in the sodium channel gene, *Scn8a,* was shown to produce viable animals with only 10% of correctly spliced mRNA in one genetic background, whereas a reduction to 5% of the correctly spliced mRNA in a different genetic background was lethal [46]. In this case, the cause of lethality was shown to be due to a mutation in the modifier gene, *Scnm1.* Furthermore, rescue from lethality was achieved by transgenic expression of a wild-type form of *Scnm1,* which brought about a 5% increase in the correctly spliced Scn8a mRNA. In another example, a bifunctional antisense oligonucleotide was introduced into fibroblasts derived from spinal muscular atrophy patients in an attempt to reprogram the splicing of exon 7 of SMN2 pre-mRNA. The SMN (survival of motor neurons) protein is a key component of nuclear gems, which are required for the biogenesis of small nuclear ribonucleoproteins. In the presence of the bifunctional oligonucleotide, exon 7 inclusion increased from 57% to 84%, and the percentage of gem-positive nuclei increased accordingly from 2%–3% to 13% [47]. Thus, subtle changes in splicing can be biologically important.

In this study, E9 of the GABA_A_ receptor γ2 subunit transcript showed an increase in exon inclusion in response to neuronal excitation, clearly opposite to that of the CI cassette. Based on the distinctive features of these two splicing control mechanisms at the biochemical level (E9 involves silencing polypyrimidine tract binding protein), the opposite responses to excitation may be due to modifications of splicing factors that are unique for each exon. Alternatively, hnRNP A1 may be involved indirectly in the regulation of E9 inclusion. At the level of cellular function, GABA_A_ receptors are the principal mediators of inhibitory neurotransmission, and the γ2 subunit plays important roles in clustering of GABA_A_ receptors and the scaffold protein gephyrin at postsynaptic sites [48,49]. Although the E9 region was originally reported to confer ethanol sensitivity to the GABA_A_ receptor [50,51], no discrete function for this region of the protein has been revealed in transgenic mice expressing the γ2S (−E9), but not γ2L (+E9) subunit isoform [52]. Thus, the possibility that the changes in splicing we observe here for GABA_A_ receptor γ2 could be related to the adaptive response of neurons to hyperstimulation is purely speculative.

Future work will be needed to describe the full spectrum of exons that respond to neuronal excitation genome-wide, and the splicing factors and signaling pathways involved. Although this silencing motif code is relatively rare, well over 100 human exons contain two or more UAGGs that could potentially confer responsiveness in neurons. This also raises the question of whether the failure of cells to reverse acute splicing responses to environmental stimuli or stress may play roles in the misregulation of splicing that contributes to disease. It will also be of interest to explore the relationships between induced splicing effects and the function and distribution of relevant neurotransmitter receptors at the synapse.

## Materials and Methods

### Plasmid construction.

Fluorescent reporters: CaMKII_EYFP reporters were constructed by inserting the open reading frame of EYFP and poly(A) site downstream of the 8.5 kb SalI-NotI fragment of the mouse CaMKII promoter [26] in a pBlueScript (pBS) plasmid. Promoter deletions were obtained by cleavage with FspI as shown in Figure 2. CaMKII-Cre promoter constructs were generated from corresponding Cre fusions, which were the generous gift of Joe Tsien and Zhenzhong Cui. Construction of Gfa2-DsRed involved inserting a 2.2-kb EcoRI fragment with the Gfa2 (glial fibrillary acidic protein) promoter upstream of the coding sequence of DsRed2 (red fluorescent protein) and downstream SV40 poly(A) site. The latter fragment was amplified by PCR from pDsRed2 (BD Biosciences, San Diego, California, United States).

Splicing reporters: Wild-type (wt0) and mutant CI cassette splicing reporters originally described in [29] were subcloned into promoter-specific plasmids CaMKII_22 or Gfa2. DIP13 splicing reporters were generated by insertion of the human DIP13 beta exon 5 and flanking introns into the NdeI and XbaI sites of the SIRT1a plasmid, followed by transfer of the HindIII-NotI fragment containing the complete splicing reporter sequence downstream of the CaMKII promoter. The exon 5 insert was synthesized in two parts, as two double-stranded deoxyoligonucleotides, and these were ligated together via complementary EcoRI cohesive ends. Site-directed mutations were generated with the QuikChange Site-Directed Mutagenesis Kit (Stratagene, La Jolla, California, United States) Sequences of all splicing reporters were verified by DNA sequencing. Constructs pBS_E18, and pBS-M3_E18 were generated by PCR from the corresponding full-length splicing reporters and cloned into the HindIII site of pBluescript vectors. All inserts were verified by DNA sequencing.

### Primary cultures, KCl treatment, and fluorescence imaging.

Approximately one dozen rat embryonic day 17 cerebral cortex tissues (Zivic Miller, Pittsburgh, Pennsylvania, United States) were treated with trypsin (0.25% trypsin, w/v, in 0.1% glucose, 2 mM L-glutamine and 20 mM HEPES in MEM) at 35 °C for 20 min. Cells were dissociated by passing the treated samples five to six times through an 18-gauge needle. The cell suspension was washed twice with ice-cold growth medium (0.5 mM L-glutamine, 2% B27 supplement in Neurabasal Medium; Invitrogen, Carlsbad, California, United States), and plated in poly-D-lysine–coated 6-well plates (BD Biosciences) at a density of 4–6 × 10^5^ cells/well. For antibody staining, cells were plated on poly-D-lysine–coated coverslips. L-glutamic acid (25 μM final concentration) was added to the growth medium for the first 4 d of culture. Cultures were maintained at 37 °C with 5% carbon dioxide. To induce depolarization, KCl was added directly to the growth medium at the indicated concentrations. Inhibitor compounds were obtained from Sigma (bicuculline; St. Louis, Missouri, United States), or Calbiochem (H89, KN93, MK801, AP5, and KT5720; San Diego, California, United States).

For immunostaining, cortical cultures on coverslips were fixed with 3.7% formaldehyde in PBS for 7 min at room temperature, and permeabilized with 0.1% TritonX-100 in phosphate buffered saline (PBS) for 1 min at −20 °C followed by incubation for 5 min at 4 °C. Coverslips were washed thoroughly with PBS, and blocked for 2 h at room temperature in Blocking Buffer (1% BSA, 0.1% gelatin in PBS) containing 5% normal goat serum. The neuron-specific mouse anti-NeuN (1:100 dilution) or glial-specific rabbit anti-GFAP (1:1,000 dilution) antibodies were applied to coverslips and incubated overnight at 4 °C. After three washes in incubation buffer at room temperature, TRITC- or FITC-labeled goat anti-mouse or goat anti-rabbit (Chemicon, Temecula, California, United States) secondary antibodies (1:1,000 dilution) were applied. After washing in Blocking Buffer (three times for 5 min each) at room temperature, coverslips were dried and mounted on slides in 10% MOWIOL 4–88 (EMD Biosciences, San Diego, California, United States), 25% glycerol, 2.5% DABCO (Sigma), and 0.1 M Tris-HCl (pH 8.5). The number of cells with EYFP and TRITC fluorescence, or the number of cells with DsRed and FITC fluorescence, were scored by confocal microscopy (Radiance 2000; Bio-Rad, Hercules, California, United States) with FITC/EYFP (460–500 nm) or TRITC/DsRed (530–550 nm) excitation filters.

### Transfection and RNA analysis.

Plasmid DNA (1.25 μg) was transfected into cortical cultures using 3 μl of Lipofectamine 2000 (Invitrogen) per well of a 6-well plate. Plasmid DNA and Lipofectamine were each diluted to a final volume of 100 μl with OPTI-MEM, then combined and incubated for 20 min at room temperature. Cultures were prepared for transfection by washing once with OPTI-MEM, followed by addition of 2-ml fresh OPTI-MEM and transfection mixture to each well. Medium was replaced with fresh growth medium after 5 h.

For splicing pattern measurements, cells were harvested 24–48 h post-transfection using TRIZOL (Invitrogen). Splicing patterns were measured in triplicate by RT-PCR as described [39]. Reverse transcription (RT) reactions (20-μl total volume) contained M-MLV reverse transcriptase (Invitrogen), 2-μg RNA sample, and 0.5-μg random hexanucleotide primers (Promega). PCR reactions (10-μl volumes) contained 0.2 μM specific primers, 2 units of Taq DNA polymerase (Promega, Madison, Wisconsin, United States), 1/20th of the volume of the RT reaction, 0.2 mM dNTPs, and 1-μCi of [α-^32^P-dCTP]. Amplification was for 25 cycles. Primers were designed to amplify exon-included and -skipped mRNAs in each sample. Primers NR1 3021 (5′) ATGCCCGTAGGAAGCAGATGC (3′) and NR1 3255 (5′) CGTCGCGGCAGCACTGTGTC (3′) were used amplify endogenous C1 cassette mRNAs. Primers rp1 (5′) GTATGGTACCCTGCACTATTTTGTG (3′) and rp2 (5′) TTGGATCGTTGCTGATCTGGGACG (3′) were used to amplify the GABA_A_ receptor γ2 mRNAs. CaMKII- and Gfa2-specific mRNAs were amplified with promoter-specific upstream primers: CaMKII, (5′) GCACGGGCAGGCGAGTGG (3′); Gfa2 (5′) TTGGAGAGGAGACGCATCACCTCC (3′) together with the gene-specific downstream primer. PCR products were resolved on 6% polyacrylamide/7 M urea gels. Gel images were captured with a BAS-2500 Phosphorimager (Fuji Medical Systems, Roselle, Illinois, United States), and quantitation determined with Science 2003 ImageGauge 4.0 software.

### Nuclear extract preparation, UV crosslinking, and affinity chromatography.

Scaled-down nuclear extracts were prepared essentially as described for rat cortical tissue [53]. Cortical cultures (five 10-cm dishes equivalent to 2 × 10^7^ cells total) were harvested using plastic cell lifters (Corning, Corning, New York, United States) in 1× PBS (1 ml/dish). After centrifugation, cell pellets were resuspended in 4 ml of ice-cold Buffer I (10 mM Tris-HCl [pH 8.0], 0.32 M sucrose, 3 mM CaCl_2_, 2 mM Mg-acetate, 0.1 mM EDTA, 0.5% NP40, 1 mM DTT, and 1× protease inhibitor cocktail). Cell suspensions were transferred to pre-chilled 15-ml Wheaton glass homogenizers, and cells were broken on ice with pestle A (tight fitting) three to five times every 10 min for a total of 30 min. Cell lysates were mixed with 4 ml of Buffer II (10 mM Tris-HCl [pH 8.0], 2 M sucrose, 5 mM Mg-acetate, 0.1 mM EDTA, 1 mM DTT, and 1× protease inhibitor cocktail), and layered on top of 3.5 ml of Buffer II in 12-ml ultracentrifuge tubes. Samples were centrifuged in a SW41 rotor at 30,000 *g* for 45 min at 4 °C. Nuclear pellets were rinsed twice with 0.5 ml of Buffer C (20 mM Hepes [pH 7.6], 20 mM KCl, 1.5 mM MgCl_2_, 25% v/v glycerol, 0.5 mM DTT, 0.2 mM EDTA), resuspended in 50-μl Buffer C plus 0.25 M KCl, and transferred to 1.5-ml Eppendorf tubes. The concentration of nuclei in each sample was determined by trypan blue staining and adjusted with buffer to equal concentrations. Nuclear suspensions were incubated for 30 min on ice with occasional mixing, then centrifuged at 14,000 rpm for 10 min at 4 °C. Nuclear extracts (50 μl volumes) were collected and used for UV crosslinking experiments. Protein concentrations were typically in the range of 0.5 to 0.75 mg/ml as determined by Bradford assay.

Short RNA substrates (22-mers) were synthesized by Dharmacon and 5′ end labeled with γ-^32^P-ATP and T4 polynucleotide kinase. Longer RNAs were internally labeled with α^32^P-UTP during in vitro transcription using T7 RNA polymerase (Stratagene). RNA-protein complexes were assembled for 5 min at 30 °C in 12.5-μl reactions by combining labeled RNA substrates (100,000 disintegrations/min [dpm]) and nuclear extract (3.5 μl) under splicing conditions (20 mM Hepes [pH 7.5], 1.5 mM ATP, 5 mM creatine phosphate, 2 mM MgCl_2_, and 4.5 μl of 4.5 λ Buffer (25% glycerol v/v, 0.02 M Hepes [pH 7.5]). Samples were irradiated on ice with 1.8 joules at 365-nm wavelength. Samples were positioned at a distance of 8 cm from the UV light source. After adding SDS-PAGE loading buffer (volume), samples were boiled 5 min at 95 °C and resolved by electrophoresis on SDS-PAGE (10% polyacrylamide separating gels). Gels were fixed in 45% methanol, 9% acetic acid for 2 h, and dried. Radioactive images were captured as described above. For affinity selection experiments, the CI cassette exon (variant E18) was cloned upstream of the M3 hairpin and synthesized by in vitro transcription by T3 RNA polymerase. Transcripts were purified on Sephadex G50–150 spin columns and ethanol precipitated. MS2-MBP was purified as described [30].

## Supporting Information

Figure S1 Depolarization Response of Endogenous GABAA Receptor Gamma 2 Exon 9, and Verification of CaMKII Reporter Expression in Neurons (A) The depolarization response of endogenous GABAA receptor gamma 2 exon 9.
(B) Verification of CaMKII reporter expression in neurons.
(852 KB TIF)

Figure S2 Immunoprecipitation Analysis of hnRNP A1, and Effect of H89 Inhibitor on UV Crosslinking of A1 Combined UV crosslinking reactions equivalent to sample shown in lane 2 of Figure 6A were used for immunoprecipitation analysis of hnRNP A1 (lanes 11–15). Arrowhead indicates position of hnRNP A1. Effect of H89 inhibitor on UV crosslinking of hnRNP A1 is shown (lanes 16–18).
(210 KB TIF)

Figure S3 Effects of Antagonists of AMPA/Kainate Receptor and Voltage-Gated Calcium Channels (L- and N-Type) on Induced Splicing of the CI Cassette Exon (10.9 MB TIF)

## Abbreviations

CaMK: calcium/calmodulin kinase
CMV: cytomegalovirus immediate early
EYFP: enhanced yellow fluorescent protein
GFAP: glial fibrillary associated protein
kb: kilobase
PKA: protein kinase A
UV: ultraviolet
